# Reply to C. Ferreira-Pêgo’s Letter to the Editor Re: Nissensohn M. et al.; *Nutrients* 2016, *8*, 232

**DOI:** 10.3390/nu8110700

**Published:** 2016-11-04

**Authors:** Mariela Nissensohn, Lluis Serra-Majem

**Affiliations:** 1 Research Institute of Biomedical and Health Sciences, University of Las Palmas de Gran Canaria, Las Palmas de Gran Canaria 35016, Spain; mnissensohn@acciones.ulpgc.es; 2 CIBEROBN, Biomedical Research Networking Center for Physiopathology of Obesity and Nutrition, Carlos III Health Institute, Madrid 28029, Spain

Dear Editor,

We would like to thank you for the opportunity to answer to the Letter to the Editor from Ferreira-Pêgo, Babio and Salas-Salvadó [1] regarding our recent publication entitled “Beverage Consumption Habits and Association with Total Water and Energy Intakes in the Spanish Population: Findings of the ANIBES Study” [2].

In reply to their comments, we would like to mention our involuntary failure to acknowledge their articles, entitled “Fluid intake from beverages in Spanish adults: Cross-sectional study” [3] and “Fluid intake in Spanish children and adolescents; a cross-sectional study” [4]; and in consequence, for not including them as part of the reviewed references in our article because we were not acquainted with their work.

However, now that they have been brought to our attention, there are few issues that deserve further comment.

The article by Ferreira-Pêgo et al., conducted in adults [3], as well as the one by Fernández-Alvira et al., conducted in a pediatric setting [4], share a common methodology and both are pieces of well-structured research. However, after considering their data, we still believe that the existing scientific literature in Spain, looking at total water intake of the population, is both poor in quantity and quality.

From our point of view, these papers require certain refinements and considerations:

First of all, they do not measure water intake coming from foods, although they did consider it when comparing water intake with EFSA recommendation after subtracting 20%, considering such percentage as an average standard for water content in foods. Even if these mathematically obtained values are theoretically precise, the fact is that, in reality, they only contribute to further increase the already existing bias. Water from foods may change from 20% to 30%, depending on various factors, such as season of the year, population income, and so on, and may vary from one individual to the other.

Secondly, as pointed out by the authors, although sufficient in size, their population sample does not fully represent the Spanish population, posing a clear limitation when considering their findings. In the pediatric article [4], the authors noted that “… As the participants were recruited as being part of a database, only individuals having telephone numbers were included. Therefore, low socioeconomic groups could be underrepresented…” whereas, in the adult study [3], “…The main limitation is that our population is probably not representative of the general Spanish population because individuals were randomly recruited from a database of volunteers for population surveys…”.

Another reason to consider is that both the study by Ferreira-Pêgo et al. [3], as well as that of Fernández-Alvira et al. [4], employed no validated beverage questionnaires. To our knowledge, the first validated questionnaire developed in Spain was created together with our research group in 2015, and the publication of which only recently appeared [5].

Lastly, it is important for us to comment that, once we took all data provided by Ferreira-Pêgo et al. [1] into consideration, they do not change the conclusions we reached in our article; furthermore, they clearly reinforce the paramount need for producing high-quality research, focusing on population water intake (from beverages and foods) with an adequate and strict scientific methodology.

We failed to identify these two studies due to the way in which references for our paper were searched for and selected. We only looked for a limited number of international journals. However, we are aware now that in future research we should include other local journals as well. Our main interest is to gradually increase the general interest placed on this emerging and fascinating area of study.
